# A real-time *ex vivo* model (eIBUB) for optimizing intraperitoneal drug delivery as an alternative to living animal models

**DOI:** 10.1515/pp-2019-0017

**Published:** 2019-08-15

**Authors:** Iaroslav Sautkin, Wiebke Solass, Frank-Jürgen Weinreich, Alfred Königsrainer, Martin Schenk, Karolin Thiel, Marc A. Reymond

**Affiliations:** National Center for Pleura and Peritoneum, University of Tübingen, Tübingen, Germany; Institute of Pathology, University of Tübingen, Tübingen, Germany; University of Tübingen, Tübingen, Germany; Department of General and Transplant Surgery, University of Tübingen, Tübingen, Germany; Experimental Surgical Oncology, Eberhard-Karls-Universitat Tubingen Medizinische Fakultat, Tübingen, Germany

**Keywords:** aerosol, alternative to animal models, cisplatin, doxorubicin, intraperitoneal chemotherapy, pressure, Pressurized Intraperitoneal Aerosol Chemotherapy (PIPAC)

## Abstract

**Background:**

Optimization of intraperitoneal drug delivery systems requires functional models. We proposed the Inverted Bovine Urinary Bladder Model (IBUB), but IBUB does not allow repeated measurements over time and there is a significant biological variability between organs.

**Methods:**

A further development of IBUB is presented, based on the physical principle of communicating vessels. Fresh bovine bladders were inverted so that the peritoneum lines up the inner surface. The IBUB and a second vessel were then interconnected under the same CO_2_ pressure and placed on two scales. The therapeutic solution (Doxorubicin 2.7 mg and Cisplatin 13.5 mg) was delivered via an aerosolizer. All experiments were in triplicate and blinded to the origin of samples, measurements in a GLP-certified laboratory.

**Results:**

The enhanced IBUB (eIBUB) model allows measurements of tissue drug concentration, depth of tissue penetration and spatial distribution. The homogeneous morphology of the peritoneum enables standardized, multiple tissue sampling. eIBUB minimizes biological variability between different bladders and eliminates the bias caused by the liquid collecting at the bottom of the model. Concentration of doxorubicin in the eIBUB (mean ± STDV: 18.5 ± 22.6 ng/mg) were comparable to clinical peritoneal biopsies (19.2 ± 38.6 ng/mg), as was depth of drug penetration (eIBUB: mean (min-max) 433 (381–486) µm, clinical ~ 500 µm).

**Conclusions:**

The eIBUB model is a simple and powerful *ex vivo* model for optimizing intraperitoneal drug delivery and represents an attractive alternative to animal models. Results obtained are similar to those obtained in the human patient.

## Introduction

Peritoneal metastasis of gastrointestinal and gynecological origin are frequent with an estimated worldwide incidence of 1,000,000 new cases/year [1, 2]. In spite of recent progress in multimodal therapy including chemotherapy, surgery and immunotherapy, prognosis is dismal and peritoneal metastasis is still perceived as a terminal condition [3]. Intraperitoneal chemotherapy offers new hope since it has been shown to improve prognosis in selected indications, both in the adjuvant and palliative situation [4]. However, intraperitoneal chemotherapy has several pharmacological limitations, in particular poor tissue penetration and incomplete distribution of the drug within the peritoneal cavity [5].

To our knowledge, there is no drug currently on the market that has been approved for intraperitoneal delivery by US FDA or European EMA. Drugs administered into the peritoneal cavity are approved in the specific indications for intravenous delivery. Thus, they can only be administered “off-label”. Importantly, these drugs have all been formulated for optimized intravenous delivery and are therefore hydrophilic [6]. This is indeed a strong limitation for application into a cavity limited by peritoneal membrane, which is covered by the glycocalyx mainly consisting of sphingolipids [7]. So far, there is no drug available in clinical practice that has been formulated specifically for the intraperitoneal administration.

Several drug delivery systems are available for administering drugs into the peritoneal cavity. Intraperitoneal catheters are the traditional way to administer intraperitoneal chemotherapy. Insertion of the catheter(s) require a surgical intervention under general anesthesia. A large volume of therapeutic solution (usually 1–3 L) is infused into the abdominal cavity with an exposition time of several hours. Then, the solution is removed through the same catheter(s). Catheter-based intraperitoneal chemotherapy is limited by a dose-dependent local and systemic toxicity and catheter complications are not rare. Moreover, the procedure is cumbersome for the patient and relatively costly [8].

Over the last three decades, Paul Sugarbaker and others have developed an approach combining cytoreductive surgery (CRS) with Hyperthermic Intraperitoneal Chemotherapy (HIPEC) [9]. CRS and HIPEC have been shown to be feasible and to prolong survival in selected patients in good condition with limited peritoneal disease and less aggressive tumor biology [10]. However, the incremental gain of HIPEC over CRS alone might be fairly limited [11, 12] probably because of the limited tissue penetration of the drugs [13].

A recent development of intraperitoneal chemotherapy is Pressurized Intraperitoneal Aerosol Chemotherapy (PIPAC). During a staging laparoscopy, the chemotherapy solution is aerosolized under pressure into the peritoneal cavity. The procedure has superior pharmacological properties [14]. PIPAC has been shown to be feasible and safe. Objective response rates and potential positive impact on quality of life were encouraging. Therefore, PIPAC can be considered a treatment for refractory, isolated peritoneal metastasis of various origins. Further indications need to be validated by ongoing prospective studies [15]. Further developments of PIPAC, such as electrostatic loading and precipitation of the therapeutic aerosol, have been proposed that could further improve the pharmacological performance of the procedure [16, 17].

In order to investigate and develop the full potential of these new systems for intraperitoneal drug delivery, there is a need for adequate functional models. For example, spatial distribution of the therapeutic aerosol needs further optimization [18]. Other needs are optimizing contact time between the therapeutic substance and the target tissue and determining the earliest time point for termination of the procedure without loss of efficacy.

Two years ago, we have proposed a new *ex vivo* model for optimizing distribution of therapeutic aerosols: the (inverted) bovine urinary bladder (IBUB) [19]. The IBUB model is easy to use, reproducible and cost-effective. It has a volume similar to the human abdominal cavity, and an oval shape. The inner surface is lined with peritoneum. The IBUB model allows pharmacological and biological analysis, including histology. However, the IBUB model does not allow repeated measurements over time, so that a large number of bladders have to be exposed to the therapeutic agent at various time points. This induces indeed a biological variability between the different organs, and this biological variability can impair analysis of smaller differences in tissue uptake or depth of tissue penetration.

In this work, we present a novel *ex-vivo* model allowing optimization of intraperitoneal drug delivery with repeated measurements over time without the need for sacrifice of additional animals.

## Materials and methods

### Design

This is an experimental, *ex-vivo* study in the IBUB model.

### Ethical and regulatory background

The research conducted did not employ live animals or human-derived specimens. No animal was sacrificed for this study. According to German law, no authorization of the Institutional Review Board or of the Animal Protection Committee was required for performing this research.

### Inverted bovine urinary bladder model

The IBUB model has been described elsewhere [20] and is established in our research laboratory. The IBUB model is well suitable for optimizing drug delivery systems targeting the peritoneum. The inflated bovine bladder has a volume (3–5 l) similar to the human abdominal cavity. The bovine urinary bladder is situated intraperitoneally and thus almost completely lined up by peritoneum. When the urinary bladder is inverted (outside-in), the inside lumen is covered by a homogeneous peritoneal layer. Fresh bovine urinary bladders were obtained from the slaughterhouse and transported to our laboratory at a temperature of 4–8 °C. The organs were thoroughly rinsed with water. Then, the organs were prepared as follows: a 2 cm incision was made in the bladder neck and the organ was carefully inverted through said incision. Then, a 12 mm balloon trocar (Kii^®^, Applied Medical, Düsseldorf, Germany) was inserted in the open bladder neck, fixed tightly with a Mersilene^®^ pursuing suture and secured by inflating the trocar’s balloon.

### Real-time measurement of weight

In order to simplify data capture, we developed an automatized electronic system for continuous measurement of IBUB weight and precipitated aerosol. Data generated by two electronic scales (Kern and Sohn GmbH, Blaingen, Germany) were transferred via DB-9 standard cable to the VGA interface of a personal computer equipped with dedicated software (Balance Connection Version 4.2.4, SCD-4.0-Pro, Kern and Sohn GmbH, Blaingen, Germany). The system allows real time weight recording (every 5** **s) from both scales and electronic data processing, including graph generation.

### Preanalytical sample preparation

Before analysis, lyophilized pellets were rehydrated and homogenized using an automatic homogenizer (TissueLyser LT, QIAGEN, Hilden, Germany). The TissueLyser LT® (ref) provides rapid and standardized, simultaneous disruption of up to 12 various tissue biopsies without risk of external contamination. Tissue disruption and homogenization is obtained through combined beating and grinding effects of beads on the sample material. Samples were placed into 2 mL ceramic tubes (Ceramic Bead Tubes Kit 2.8 mm diameter, Artnr. 13114-50, QIAGEN, Hilden, Germany) with the ceramic beads inside and rehydrated with 1.5 mL of sterile distilled water (Ampuwa, Fresenius KABI, Homburg, Germany). Then the ceramic tubes were placed into the TissueLyser LT and shaked a high frequency (50 Hz, 3000 oscillations/min) in a vertical position for 1 h at RT. In addition, the ceramic tubes can be placed in a ultrasound device (ELMA, S30H Elmasonic, Singen, Germany) and allowed to keep in the activated ultrasound device for 10 min at RT. Then the ceramic tubes were vortexed for 30 s and centrifuged for 10 min (Eppendorf, centrifuge 5417R, 9000 rpm; Hamburg, Germany) at RT.

### Depth of tissue penetration

Deep-frozen biopsies were embedded in TissueTek (Tissue-Tek, Sacura REF 4583) and cut into 10 μm slices in a cryotome (Leica cryocut CM3050S, Leica, Nussloch, Germany). Depth of tissue penetration was determined by measuring spontaneous fluorescence of using a Name microscope (Leica DMRBE, Wetzlar, Germany). Depth of tissue penetration was assessed by spontaneous fluorescence of doxorubicin with a fluorescence microscope (Leica Quantimed Q 600 with filter: doxorubicin ex 490 nm abs 560, 590 nm) (magnitude 10x). Results were documented with Leica Qwin 2002 software. Measurements were performed in triplicate by an independent pathologist (W.S.) blinded to the identity of the samples.

### Drug tissue concentration

Measurements were performed in triplicate. Biopsies were sent to an independent, GLP-certified laboratory (Labor Eberhard, Dortmund, Germany). The laboratory was blinded to the sample identity. Doxorubicin concentration was measured by high performance liquid chromatography (HPLC; Waters Fluorescence Detector 2475, Waters Inc., Milford, MA), with a serum LLoQ of 5 ng/mL. Pre-analytical validation proved a linear range of measurements in 5% glucose matrix between 0.1 and 10,000 µg/mL doxorubicin and established no influence of organic matrices. Cisplatin was quantified by atomic absorption spectroscopy (AAS; ZEEnit P 650, Analytic Jena AG, Jena, Germany). The lower level of quantification (LLoQ) for platinum was 50 ng/mL (cisplatin 80 ng/mL; calculation factor 1.54). Pre-analytical validation proved a linear range of measurements in 5% glucose matrix between 0.1 and 100 µg/mL platinum, and established no influence of organic matrices.

### Occupational health safety

This research involved chemotherapeutic, toxic substances with a potential health risk for the personal involved. The research laboratories of NCPP have been audited successfully in fall 2016 for compliance with German law and safety guidelines. The personal has been trained and access to the facilities is restricted. All experiments with toxic substances are performed within a Class 3 safety hood (Maxisafe 2000, ThermoFisher Scientific, Dreieich, Germany) certified for handling of cytotoxic substances. Air analysis for potential platin contamination were performed under working conditions in November 2016 by an external certification company (DEKRA industrials, Stuttgart, Germany) and revealed no traces of platin. Surface contamination is monitored at regular intervals. The personal involved is regularly monitored by a physician specialized in occupational medicine.

### Data management and statistics

Experimental data were documented according to Good Scientific Practice and uploaded using LabGuru software (BioData Inc., Cambridge, MA, USA). Statistical calculations were performed with SPSS statistics software, version 25 (IBM Inc, Armonk, NY, USA). Comparison of means was performed using non-parametric tests (Mann-Whitney U-test).

## Results

In prior work, we have described an innovative model for optimizing homogeneity of drug delivery using therapeutic pressurized aerosols, the IBUB model [20]. Fresh bovine urinary bladder *ex vivo* can be used for evaluating the target effect of therapeutic aerosol both onto the mucosa or, by inverting the organ, onto the serosa. IBUB is a novel *ex-vivo* model in which physicochemical characteristics of a therapeutic aerosol can be optimized easily.

However, we found that IBUB had limitations, for example for determining tissue drug uptake over time. Dynamic experiments required a large number of bladders to perform repeated biopsies. Moreover, when comparing these different bladders, we faced some biological variability between organs, depending for example on weight, wall thickness and hydration state of the tissue.

In order to determine reasons for results variability between various IBUB experiments, the organs were examined by histopathology. As exemplified in Supplemental Material, Figure 1, the fresh, cooled organs were typically well preserved and no sign of tissue necrosis was observed.

In the meantime, based on our experience, we routinely sample a biopsy from the bladder neck before experiments in order to verify tissue integrity by HE staining.

Then, we compared tissue drug uptake between various organs. Results can be seen from Supplemental Material, Figure 2. Whereas this variability did not reach statistical difference for doxorubicin (ANOVA, p=0.52), it was significant for cisplatin tissue concentration (ANOVA, p<0.05).

**Figure 1:**
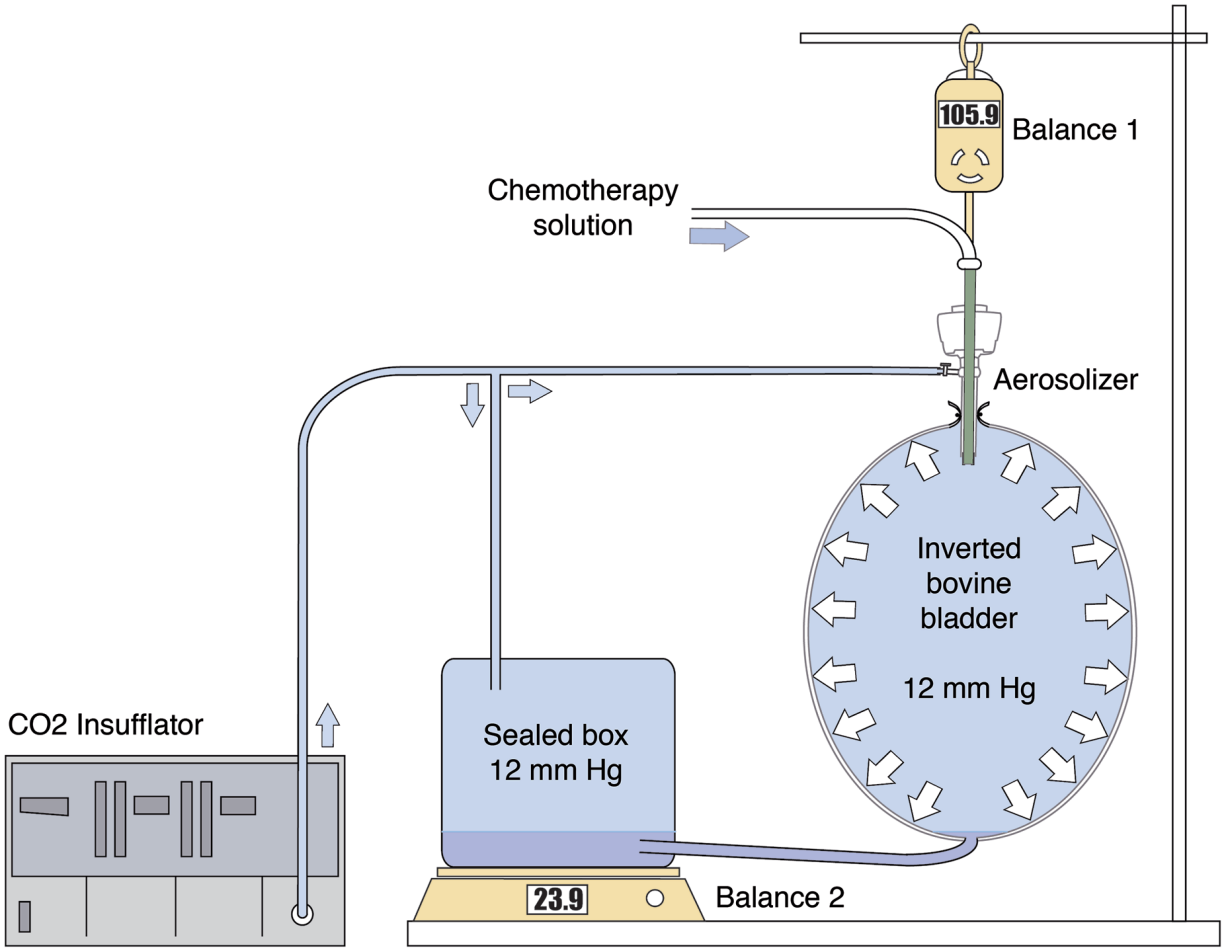
Principle of the enhanced Inverted Bovine Urinary Bladder (eIBUB) model. The inverted urinary bladder is interconnected with a second container and the weight of both vessels are measured in real time.

The variability observed in the results between different organs convinced us to use a single organ for repeated measure, which required further development of the IBUB model. The principle of the enhanced IBUB (eIBUB) model is displayed in Figure 1. This model relies on the principle of communicating vessels. Two vessels (the IBUB itself and a separated, closed plastic contained) are interconnected tightly with a silicone tubing sewed at the bottom of the IBUB, creating a closed system. Each vessel is placed on an individual balance (Balance 1 or Balance 2). Additionally, both vessels are filled with CO_2_ at the same pressure (e. g. 12 mmHg but this pressure can be modified).

**Figure 2:**
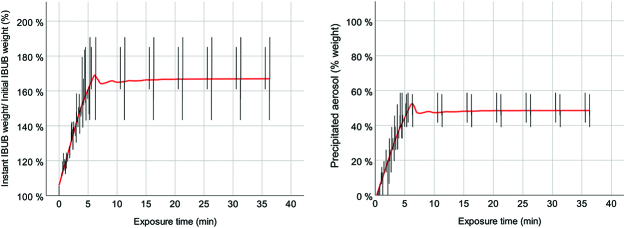
Dynamic measurements over time. Left panel: Changes in weight of the IBUB. Right panel: weight of the aerosol precipitating over time.

This design allows real-time weight measurement of the IBUB and the second vessel. The weight gain (δW bladder) of the IBUB and of the vessel (δW vessel) reflect in real-time the liquid uptake into the tissue, and the precipitating aerosol, respectively.

We compared the reproducibility of tissue drug concentration of cisplatin and doxorubicin between the IBUB and the eIBUB model. As Table 1 is showing, differences in drug concentration were larger when multiple biopsies (n=12–42) from different bladders vs. biopsies from the same bladder (n=23) were compared.

**Table 1:** Variability of measured Cisplatin and Doxorubicin concentrations between urinary bladders (IBUB model) and within a single urinary bladder (eIBUB model).

	Between urinary bladders				Same urinary bladder			
Position	Cisplatin	Sign.	Doxorubicin	Sign.	Cisplatin	Sign.	Doxorubicin	Sign.
Top	17.5 (13.6–21.4)	0.001*	0.7 (0.4–1.0)	0.10	15.0 (9.7–20.3)	0.77	0.8 (0.3–1.2)	0.25
Middle	7.3 (4.5–10.0)	0.002*	0.3 (0.0–0.6)	0.04*	12.8 (7.5–18.1)	0.94	0.4 (0.2–0.7)	0.93
Bottom	7.0 (3.5–10.6)	0.06	0.3 (0.1–0.4)	0.03*	12.8 (8.2–17.5)	0.87	0.4 (0.2–0.7)	0.63

Mean; CI 5–95%. ANOVA; *significant difference (p<0.05). Values (in ng/mg) are normalized to allow comparison.

In Figure 2 we compare the weight of the IBUB with the aerosol precipitating over time in form of a liquid at the bottom of the bladder. The weight gain of the bladder increases by 60–70% within 5–10 min. As we can see, a significant fluid volume is taken up rapidly into the tissue, showing evidence that PIPAC is a high volume drug delivery system. Since large-molecular weight molecules are essentially taken up by convection (and not by diffusion), PIPAC is expected to be effective in delivering large drug quantities into the subperitoneal tissue. We observe from the right panel that about half of the aerosol precipitates within a comparable time frame. The liquid collected in real time at the bottom of the urinary bladder reflects the combined effect of impaction and sedimentation forces onto the therapeutic aerosol. This volume of liquid also provides information on the adhesive properties of the aerosol onto the whole surface of the peritoneum exposed to the aerosol, since the therapeutic substance in contact with the target tissue will not drip down the lateral walls of the IBUB.

**Figure 3:**
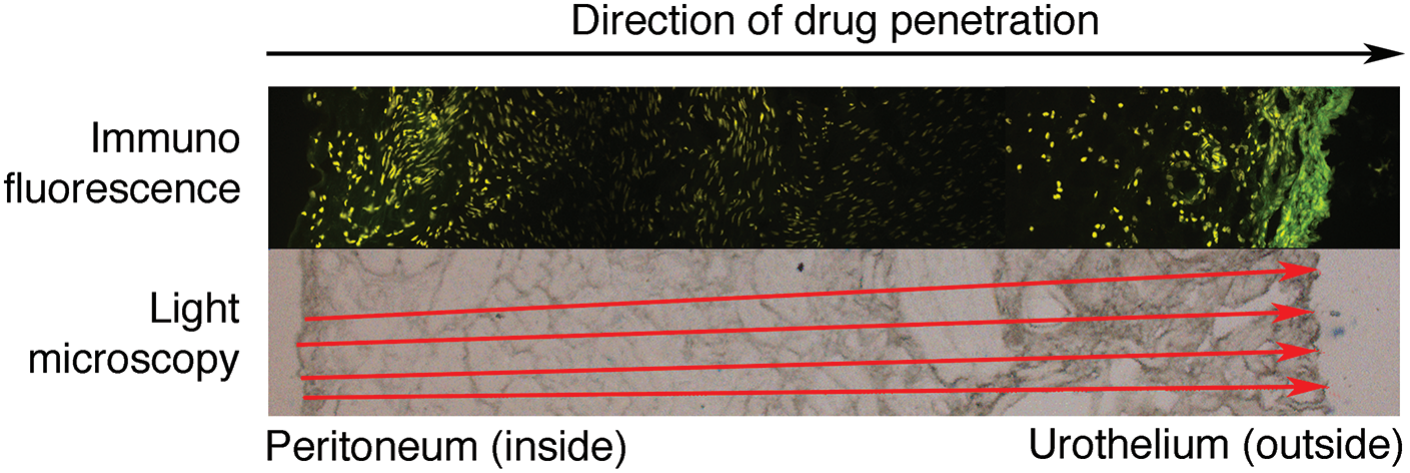
Measurement of depth of tissue penetration using fluorescence microscopy. In this example (biopsy at the bottom of the enhanced IBUB model after PIPAC), we observe a transparietal drug penetration of Doxorubicin up to a depth of 3807 ± 1290 µm. (mean ± STDV).

The enhanced IBUB model allows measurement of drug penetration into the tissue. For example, the (spontaneous) fluorescence of doxorubicin can be observed by fluorescence microscopy in Figure 3. Alternatively, it is also possible to use fluorescent labeling with DAPI, Cyanin 5, curcumin or other staining substances. For this purpose, after completion of the experiment, the bladder is opened and serial punch biopsies (standard diameter 8 mm) are taken in triplicate at the top, in the middle and at the bottom of the organ. This allows, for example, comparison of tissue uptake at different positions of the IBUB and optimization of aerosolizing devices in combination with various substances and formulations.

**Figure 4:**
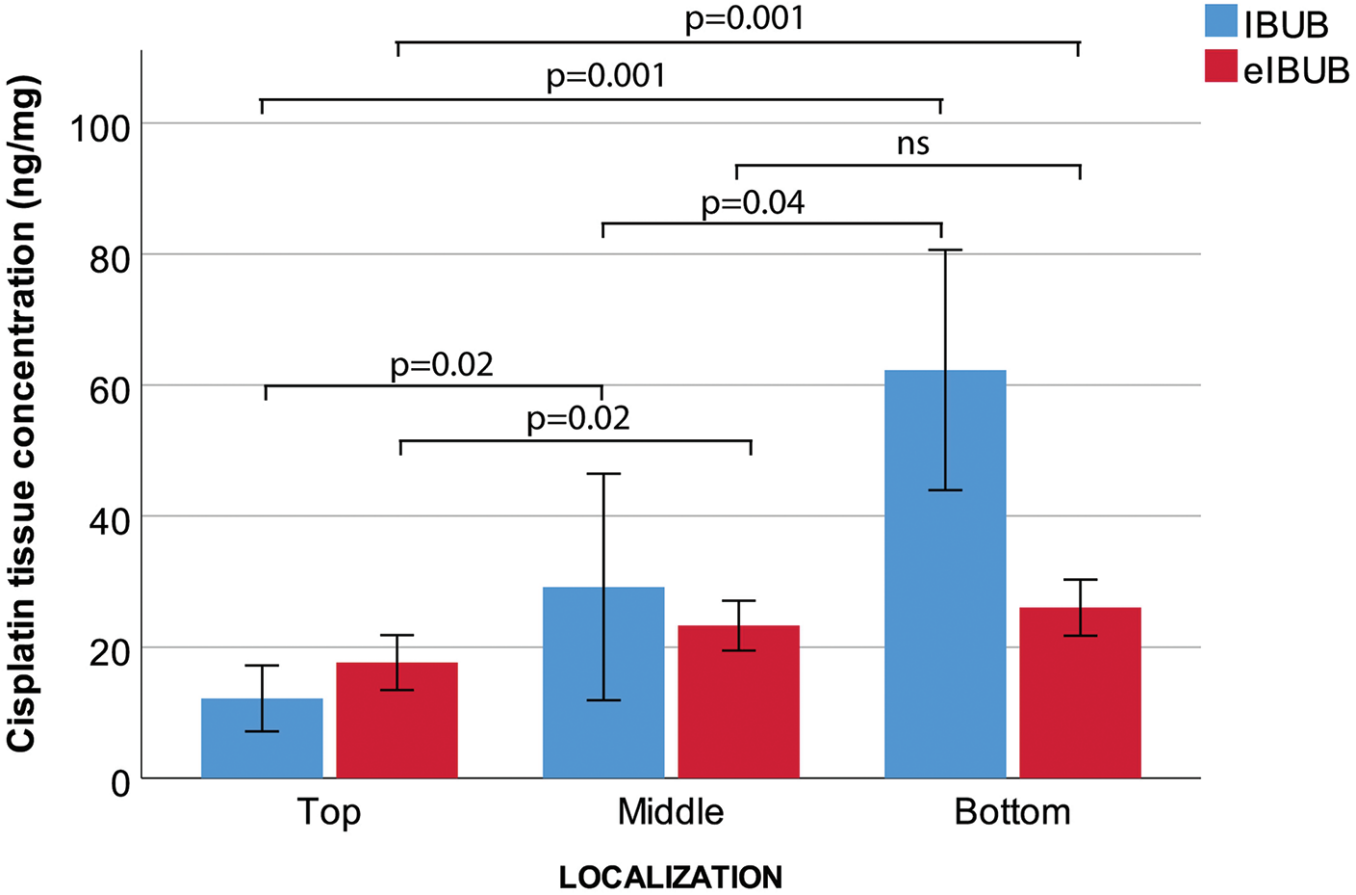
Comparison of tissue concentration of Cisplatin in the IBUB (blue) vs. enhanced IBUB model (red). Experimental conditions are the same (Aerosolization of 13.5 mg cisplatin in 150 mL NaCl 0.9% at RT and a capnoperitoneum of 12 mmHg). IBUB: Inverted Bovine Urinary Bladder. eIBUB: enhanced Inverted Bovine Urinary Bladder.

The IBUB model allows measurements of drug tissue concentration. When implementing the eIBUB model in our laboratory, we hypothesized that drug concentration might differ between the original IBUB model and the eIBUB model. Therefore, we compared the results obtained under the same experimental conditions in both models. As shown in Figure 4, a statistical significant vertical cisplatin concentration gradient was observed both in the IBUB and the eIBUB models (p=0.001). However, whereas the tissue concentration of cisplatin was comparable at the top and the middle of the urinary bladder, it was reduced at the bottom of the eIBUB as compared to the previous IBUB model. This difference in the tissue concentration of cisplatin is likely explained by the absence of liquid collection at the bottom of the experimental model in the eIBUB.

For validating the eIBUB model we compared pharmacological measurements obtained for doxorubicin delivered as PIPAC in the human patient and in the eIBUB. Results are summarized in Table 2. Since clinical biopsies are taken from the antero-lateral abdominal wall, we compared the human data with the data obtained from the superior biopsies in the eIBUB.

**Table 2:** Doxorubicin tissue concentration and depth of tissue penetration in the enhanced Inverted Bovine Urinary Bladder (eIBUB) model vs. in the human patient.

	Tissue concentration (mean ± stand. dev.)	Depth of penetration (mean, min-max)
eIBUB model		
eIBUB, n=36 biopsies in 12 bladders	18.5 ± 22.6 ng/mg	433 (381–486) µm
**Human patient**		
Solass et al. [14]	N/A	~ 500 µm
Tempfer et al. [19] (n=32 patients)	19.2 ± 38.6 ng/mg	N/A

## Discussion

Animals are used in most preclinical studies on IP drug delivery [17, 21, 22, 23, 24, 25, 26]. These animal models have been applied for improving depth of tissue penetration, homogeneity of distribution and also to optimize contact time between the therapeutic aerosol and the target tissue. However, animal models have several limitations. First, they require performance of experiments in living creatures, which implies some degree of suffering and sacrifice of animals. Second, there is a biological variability between different animals that prevent detection of small differences in experimental measures. Moreover, animal experiments are cumbersome, long and expensive. Taken together, whereas animal experiments are mandatory for preclinical pharmacological and toxicological studies, they are not well suited for optimizing therapeutic aerosols for intraperitoneal drug delivery.

Instruments for examining nature and content of aerosols have been developed as early as 1860. These instruments, generally called “impactors”, rely on a simple principle – a jet of particle-laden air impinging on a plate. In the early 1950s, Andersen proposed an impactor consisting of a cascade of microperforated plates. Dependent on their aerodynamic diameter, larger particles will impact on an early stage. Smaller particles will remain floating in the gas stream and pass to the next stage where the process starts again [27]. Along with extensive theoretical analysis of jet impaction, this impactor allowed significant progress in pulmonary aerosol medicine. However, no impactor dedicated to intraperitoneal drug delivery as pressurized aerosols is available so far.

Our aim was to develop an enhanced functional model allowing to investigate and develop the full potential of new systems for intraperitoneal drug delivery without sacrifice of animals. The urinary bladders used in this study were procured by the slaughterhouse and were obtained in cows bred for the alimentary chain. No single additional animal was sacrificed for this study.

As expected, we found that the eIBUB model allows measurement of depth of tissue penetration, drug tissue concentration and homogeneity of spatial drug distribution. Importantly, quantitative measures obtained for doxorubicin in the eIBUB model were close to measurements reported in the human patient, both for depth of drug penetration into the tissue and for tissue drug concentration. Beside these expected advantages, we found also unexpected valuable properties of the eIBUB model over available animal models. First, the (parietal) peritoneum covering the inner surface of the IBUB is homogeneous. This is in contrast with animal models, where differences in drug uptake of up to one order of magnitude have been observed between parietal (30% of the peritoneal surface mostly located anteriorly) and visceral peritoneum (70% of the animal surface, mostly located posteriorly) [28]. The eIBUB model eliminates residual liquid at the bottom of the organ and the vertical drug concentration gradient between the top and the bottom of the urinary bladder is less pronounced in the eIBUB than in the IBUB model: this is indeed important when the aim is to optimize the spatial distribution of the aerosol (as such): the collection of liquid chemotherapy solution induces a bias that needs to be eliminated. In some animal studies, liquid collections after PIPAC have been misinterpreted as a poor aerosol distribution [23].

Secondly, the geometry of the enhance IBUB is a simple ellipsoid, and aerosol repartition is not hampered by anatomical structures or adhesions [29]. This simple geometry is indeed helpful when the task is to measure homogeneity of spatial distribution of a therapeutic aerosol in a given volume. Third, the eIBUB model allows real-time measurement of tissue liquid uptake and aerosol precipitation, which has not been described in an animal model yet. Finally, the eIBUB model can be used for optimizing the contact time between the therapeutic aerosol and the peritoneum, since real-time monitoring of the liquid dripping down the serosa is possible.

The present work fits well into the research map in the field. There is a rapidly increasing number of preclinical publications evaluating PIPAC [30] for distributing various kind of therapeutic substances, including conventional chemotherapy [26], siRNA [31], siDNA [32], nanomolecules [33], antiadhesive substances [34] and even cellular therapies [35]. For each substance, there specific formulations need to be developed to optimize the contact time between the therapeutic aerosol and the peritoneum. In parallel, the potential of hyperthermia [36] and of electrostatic precipitation [17] for further optimization is currently explored. PIPAC is a generic drug delivery system and its further development is a complex process in multiple domains. In the absence of the (enhanced) IBUB model, this process would require numerous animal experiments. By providing a simple and reproducible experimental system, the eIBUB model might facilitate the task of researchers and regulatory authorities in evaluating quantitatively differences in drug concentration and/or drug tissue penetration induced by novel formulations or modified medical devices. Assuming such differences to be small demonstration of equivalence, the model could replace numerous animal experiments and/or selected clinical studies.

The eIBUB model does not allow pharmacokinetics and pharmacodynamics studies. Assessment of the biological effect of a given drug on the target tissue is not possible with this model. Even though the establishment of the eIBUB model in peritoneal drug delivery research is in our experience extremely helpful and valuable, this model will indeed not completely replace the use of living animals for developing drugs for intraperitoneal delivery. In addition, regulatory authorities usually require toxicity studies in at least two animal models preceding first in-human use [37]. Moreover, functional animal models reproducing human peritoneal disease are largely lacking, and new developments are most welcome [38].

In summary, we have presented a new functional model, the eIBUB for real-time monitoring of tissue uptake of therapeutic aerosols used in peritoneal medicine. The eIBUB model allows measurement of depth of tissue drug penetration, drug concentration, homogeneity of spatial drug distribution, liquid uptake and aerosol precipitation over time. Indirectly, it allows measurement of the contact time between a formulated therapeutic aerosol and the peritoneum. By using different therapeutic substances and physicochemical conditions, the eIBUB appears to be a powerful and generic model for further optimization of intraperitoneal drug delivery. Last but not least, eIBUB model has already saved many animal lives and is expected to save much more in the future.

## References

[1] Ferlay J, Colombet M, Soerjomataram I, Mathers C, Parkin DM, Piñeros M, et al. Estimating the global cancer incidence and mortality in 2018: GLOBOCAN sources and methods. Int J Cancer 2019;144:1941–53. 10.1002/ijc.31937 30350310

[2] Piso P, Arnold D. Multimodal treatment approaches for peritoneal carcinosis in colorectal cancer. Dtsch Arztebl Int 2011;108:802–8. 10.3238/arztebl.2011.0802 PMC3240780 22190994

[3] Lambert LA. Looking up: recent advances in understanding and treating peritoneal carcinomatosis. CA Cancer J Clin 2015;65:284–98. 10.3322/caac.21277 25940594

[4] Jaaback K, Johnson N, Lawrie TA. Intraperitoneal chemotherapy for the initial management of primary epithelial ovarian cancer. Cochrane Database Syst Rev 2016:CD005340. DOI:10.1002/14651858.CD005340.pub4. PMC8602974 26755441

[5] Dedrick RL, Flessner MF. Pharmacokinetic problems in peritoneal drug administration: tissue penetration and surface exposure. J Natl Cancer Inst 1997;89:480–7. 10.1093/jnci/89.7.480 9086004

[6] de Bree E, Michelakis D, Stamatiou D, Romanos J, Zoras O. Pharmacological principles of intraperitoneal and bidirectional chemotherapy. Pleura Peritoneum 2017;2:47–62. 10.1515/pp-2017-0010 PMC6405033 30911633

[7] Kawanishi K. Diverse properties of the mesothelial cells in health and disease. Pleura Peritoneum 2016;1:79–89. 10.1515/pp-2016-0009 PMC6386382 30911611

[8] Markman M. Chemotherapy: limited use of the intraperitoneal route for ovarian cancer-why? Nat Rev Clin Oncol 2015;12:628–30. 10.1038/nrclinonc.2015.177 26462129

[9] Sugarbaker PH. Pharmacology of chemotherapy treatments for peritoneal metastases: optimizing and augmenting HIPEC. Pleura Peritoneum 2017;2:43–5. 10.1515/pp-2017-0011 PMC6328074 30911632

[10] Lambert LA, Hendrix RJ. Palliative management of advanced peritoneal carcinomatosis. Surg Oncol Clin N Am 2018;27:585–602. 10.1016/j.soc.2018.02.008 29935691

[11] Evrard S, Mazière C, Désolneux G. HIPEC: standard of care or an experimental approach? Lancet Oncol 2012;13:e462–3. 10.1016/S1470-2045(12)70448-5 23117000

[12] Königsrainer A, Rau B. Cytoreductive surgery (CRS) and hyperthermic intraperitoneal chemotherapy (HIPEC): don’t throw the baby out with the bathwater. Pleura Peritoneum 2018;3:20180131. 10.1515/pp-2018-0131 PMC6405014 30911670

[13] Steuperaert M, Debbaut C, Segers P, Ceelen W. Modelling drug transport during intraperitoneal chemotherapy. Pleura Peritoneum 2017;2:73–83. 10.1515/pp-2017-0004 PMC6405034 30911635

[14] Solass W, Kerb R, Mürdter T, Giger-Pabst U, Strumberg D, Tempfer C, et al. Intraperitoneal chemotherapy of peritoneal carcinomatosis using pressurized aerosol as an alternative to liquid solution: first evidence for efficacy. Ann Surg Oncol 2014;21:553–9. 10.1245/s10434-013-3213-1 PMC3929768 24006094

[15] Alyami M, Hübner M, Grass F, Bakrin N, Villeneuve L, Laplace N, et al. Pressurized intraperitoneal aerosol chemotherapy: rationale, evidence and potential indications. Lancet Oncol 2019. (in press). 10.1016/S1470-2045(19)30318-3 31267971

[16] Reymond M, Demtroeder C, Solass W, Winnekendonk G, Tempfer C. Electrostatic precipitation pressurized intraperitoneal aerosol chemotherapy (ePIPAC): first in-human application. Pleura Peritoneum 2016;1:109–16. 10.1515/pp-2016-0005 PMC6386498 30911614

[17] Kakchekeeva T, Demtröder C, Herath NI, Griffiths D, Torkington J, Solaß W, et al. In vivo feasibility of electrostatic precipitation as an adjunct to pressurized intraperitoneal aerosol chemotherapy (ePIPAC). Ann Surg Oncol 2016;23:592–8. 10.1245/s10434-016-5108-4 PMC5149560 26842487

[18] Khosrawipour V, Khosrawipour T, Diaz-Carballo D, Förster E, Zieren J, Giger-Pabst U. Exploring the spatial drug distribution pattern of pressurized intraperitoneal aerosol chemotherapy (PIPAC). Ann Surg Oncol 2016;23:1220–4. 10.1245/s10434-015-4954-9 26553440

[19] Tempfer CB, Hilal Z, Dogan A, Petersen M, Rezniczek GA. Concentrations of cisplatin and doxorubicin in ascites and peritoneal tumor nodules before and after pressurized intraperitoneal aerosol chemotherapy (PIPAC) in patients with peritoneal metastasis. Eur J Surg Oncol 2018;44:1112–17. 10.1016/j.ejso.2018.04.020 29753612

[20] Schnelle D, Weinreich FJ, Kibat J, Reymond MA. A new ex vivo model for optimizing distribution of therapeutic aerosols: the (inverted) bovine urinary bladder. Pleura Peritoneum 2017;2:37–41. 10.1515/pp-2017-0006 PMC6328072 30911631

[21] Reymond MA, Hu B, Garcia A, Reck T, Köckerling F, Hess J, et al. Feasibility of therapeutic pneumoperitoneum in a large animal model using a microvaporisator. Surg Endosc 2000;14:51–5. 10.1007/s004649900010 10653236

[22] Solaß W, Hetzel A, Nadiradze G, Sagynaliev E, Reymond MA. Description of a novel approach for intraperitoneal drug delivery and the related device. Surg Endosc 2012;26:1849–55. 10.1007/s00464-012-2148-0 22580869

[23] Bellendorf A, Khosrawipour V, Khosrawipour T, Siebigteroth S, Cohnen J, Diaz-Carballo D, et al. Scintigraphic peritoneography reveals a non-uniform (99 m)Tc-Pertechnetat aerosol distribution pattern for pressurized intra-peritoneal aerosol chemotherapy (PIPAC) in a swine model. Surg Endosc 2018;32:166–74. 10.1007/s00464-017-5652-4 28643076

[24] Khosrawipour V, Khosrawipour T, Kern AJ, Osma A, Kabakci B, Diaz-Carballo D, et al. Distribution pattern and penetration depth of doxorubicin after pressurized intraperitoneal aerosol chemotherapy (PIPAC) in a postmortem swine model. J Cancer Res Clin Oncol 2016;142:2275–80. 10.1007/s00432-016-2234-0 PMC11819277 27590613

[25] Khosrawipour V, Khosrawipour T, Hedayat-Pour Y, Diaz-Carballo D, Bellendorf A, Böse-Ribeiro H, et al. Effect of whole-abdominal irradiation on penetration depth of doxorubicin in normal tissue after pressurized intraperitoneal aerosol chemotherapy (PIPAC) in a post-mortem swine model. Anticancer Res 2017;37:1677–80. 10.21873/anticanres.11498 28373428

[26] Eveno C, Haidara A, Ali I, Pimpie C, Mirshahi M, Pocard M. Experimental pharmacokinetics evaluation of chemotherapy delivery by PIPAC for colon cancer: first evidence for efficacy. Pleura Peritoneum 2017;2:103–9. 10.1515/pp-2017-0015 PMC6405032 30911638

[27] Marple VA. History of impactors – The first 110 years. Aerosol Sci Technol 2004;38:247–92.

[28] Pabst UF, Bucur P, Demtröder CR, Hölzen JP, Roger S, Tabchouri N et al. Gewebe und Blutkonzentration von Platin verabreicht mittels (e)PIPAC und L-HIPEC. 136. Kongress der Deutschan Gesellschaft für Chirurgie, München, 26–29 3 2019 (Abstract-ID:546).

[29] Solass W, Struller F, Horvath P, Königsrainer A, Sipos B, Weinreich FJ. Morphology of the peritoneal cavity and pathophysiological consequences. Pleura Peritoneum 2016;1:193–201. 10.1515/pp-2016-0023 PMC6328071 30911623

[30] Hübner M. In search of evidence - PIPAC on the fast lane. Pleura Peritoneum 2018;3:20180119. DOI:10.1515/pp-2018-0119. PMC6404989 30911660

[31] Minnaert AK, Dakwar GR, Benito JM, García Fernández JM, Ceelen W, De Smedt SC, et al. High-pressure nebulization as application route for the peritoneal administration of siRNA complexes. Macromol Biosci 2017;17:10. 10.1002/mabi.201700024 28614632

[32] Solass W, Herbette A, Schwarz T, Hetzel A, Sun JS, Dutreix M, et al. Therapeutic approach of human peritoneal carcinomatosis with Dbait in combination with capnoperitoneum: proof of concept. Surg Endosc 2012;26:847–52. 10.1007/s00464-011-1964-y PMC3271218 22042585

[33] Mikolajczyk A, Khosrawipour V, Schubert J, Grzesiak J, Chaudhry H, Pigazzi A, et al. Effect of liposomal doxorubicin in pressurized intra-peritoneal aerosol chemotherapy (PIPAC). J Cancer 2018;9:4301–5. 10.7150/jca.26860 PMC6277654 30519333

[34] Schubert J, Khosrawipour V, Chaudhry H, Arafkas M, Knoefel WT, Pigazzi A, et al. Comparing the cytotoxicity of taurolidine, mitomycin C, and oxaliplatin on the proliferation of in vitro colon carcinoma cells following pressurized intra-peritoneal aerosol chemotherapy (PIPAC). World J Surg Oncol 2019;17:93. 10.1186/s12957-019-1633-5 PMC6547564 31159819

[35] Mikolajczyk A, Khosrawipour V, Schubert J, Chaudhry H, Pigazzi A, Khosrawipour T. Particle stability during pressurized intra-peritoneal aerosol chemotherapy (PIPAC). Anticancer Res 2018;38:4645–9. 10.21873/anticanres.12769 30061231

[36] Jung Do H, Son SY, Oo AM, Park YS, Shin DJ, Ahn SH, et al. Feasibility of hyperthermic pressurized intraperitoneal aerosol chemotherapy in a porcine model. Surg Endosc 2016;30:4258–64. 10.1007/s00464-015-4738-0 26715024

[37] US FDA. Guidance for industry M3(R2) nonclinical safety studies for the conduct of human clinical trials and marketing authorization for pharmaceuticals. Available at: https://www.fda.gov/media/71542/download. Accessed: 4 May 2019. 20349552

[38] Van de Sande L, Willaert W, Cosyns S, De Clercq K, Shariati M, Remaut K, et al. Establishment of a rat ovarian peritoneal metastasis model to study pressurized intraperitoneal aerosol chemotherapy (PIPAC). BMC Cancer 2019;19:424. 10.1186/s12885-019-5658-5 PMC6503553 31064330

